# Identification of *ZNF26* as a Prognostic Biomarker in Colorectal Cancer by an Integrated Bioinformatic Analysis

**DOI:** 10.3389/fcell.2021.671211

**Published:** 2021-06-11

**Authors:** Jiaxin Liu, Yimin Li, Yaqi Gan, Qing Xiao, Ruotong Tian, Guang Shu, Gang Yin

**Affiliations:** ^1^Department of Pathology, Xiangya Hospital, School of Basic Medical Sciences, Central South University, Changsha, China; ^2^School of Basic Medical Sciences, Central South University, Changsha, China; ^3^China-Africa Research Center of Infectious Diseases, School of Basic Medical Sciences, Central South University, Changsha, China

**Keywords:** GEO, TCGA, CRC, prognosis, transcription factors, *ZNF26*

## Abstract

The dysregulation of transcriptional factors (TFs) leads to malignant growth and the development of colorectal cancer (CRC). Herein, we sought to identify the transcription factors relevant to the prognosis of colorectal cancer patients. We found 526 differentially expressed TFs using the TCGA database of colorectal cancer patients (*n* = 544) for the differential analysis of TFs (*n* = 1,665) with 210 upregulated genes as well as 316 downregulated genes. Subsequently, GO analysis and KEGG pathway analysis were performed for these differential genes for investigating their pathways and function. At the same time, we established a genetic risk scoring model for predicting the overall survival (OS) by using the mRNA expression levels of these differentially regulated TFs, and defined the CRC into low and high-risk categories which showed significant survival differences. The genetic risk scoring model included four high-risk genes (*HSF4*, *HEYL*, *SIX2*, and *ZNF26*) and two low-risk genes (*ETS2* and *SALL1*), and validated the OS in two GEO databases (*p* = 0.0023 for the GSE17536, *p* = 0.0193 for the GSE29623). To analyze the genetic and epigenetic changes of these six risk-related TFs, a unified bioinformatics analysis was conducted. Among them, *ZNF26* is progressive in CRC and its high expression is linked with a poor diagnosis as well. Knockdown of *ZNF26* inhibits the proliferative capacity of CRC cells. Moreover, the positive association between *ZNF26* and cyclins (CDK2, CCNE2, CDK6, CHEK1) was also identified. Therefore, as a novel biomarker, *ZNF26* may be a promising candidate in the diagnosis and prognostic evaluation of colorectal cancer.

## Introduction

Colorectal cancer is the third most common cancer form in the world, accounting for nearly 600,000 deaths per year (Ferlay et al., 2010; Jemal et al., 2011). Colorectal cancer develops as a result of the accumulation of genetic and epigenetic changes over time (Pritchard and Grady, 2011). It has been one of the most common causes of cancer-related deaths due to a low survival rate, notably in patients in the last stages (Zhang et al., 2017). Therefore, to improve tumor therapy and patient survival, new diagnostic, prognostic, and therapeutic biomarkers must be investigated and employed (Song et al., 2018). Routine clinical pathology risks and various prognostic aspects including age, tumor status, grade, and CEA levels determine the CRC treatment strategies and their clinical outcomes (Hsing et al., 1998; He et al., 2018).

Transcription factors are a class of proteins that bind to unique sequences upstream of a target gene’s 5′ end, which is commonly referred to as a promoter region (Beato and Eisfeld, 1997; Sikder and Kodadek, 2005). Hence, these transcription factors can obstruct or strengthen the expression of genes and assure the expression of target genes at the definite times (Eckersley-Maslin et al., 2018). Gene transcription, which regulates all cellular functions, can be controlled by epigenetic modifications. Furthermore, epigenetic modification can also occur on the gene loci encoding certain transcription factors (TFs), thus making them a new regulator of cellular functions and biological processes (Wu et al., 2016). In a normal state, basic biological activities, which include differentiation, development, and metabolism also involve the promoter-specific transcription factors (Brown and Goldstein, 1997; Kang and Malhotra, 2015). Deregulation of such transcriptional factors has been linked to the formation of cancer and its deadly progression (Cao et al., 2018).

This study aimed to learn more about the biology of TFs in CRC. Up to this level, the expression of TF, already contained in the Animal TFDB database in colorectal cancer, was analyzed. Through the GO and KEGG pathways analyses, we investigated the pathways and functions in which differentially expressed TFs may be involved. At the same time, a set of CRC-specific TFs was identified in which the TF prognostic features were obtained. By analyzing various public data sets, we claimed that a further analysis of the expression and sequence changes of these differentially regulated TFs with different regulations may help to alter the expressions in CRC. Additionally, *ZNF26* belongs to the zinc finger protein family, and we discovered that its high expression is linked to a poor prognosis. Moreover, *ZNF26* is positively associated with cyclins (CDK2, CCNE2, CDK6, and CHEK1).

## Materials and Methods

### TCGA Data Set Selection and Data Processing

The GDC Data Portal^1^ was used to collect miRNAs, mRNA data, and related clinical information from CRC patients in the TCGA project. After that, R-studio software 3.5.3 was used for examining the data for 525 tumor tissue samples and 44 normal tissue samples (Clinical information of four patients was missing). According to the pathologic stage, the number of tumor tissues in stages I, II, III, and IV were 93, 206, 148, and 78, respectively. Using the “edgeR package” of *p*-value < 0.05 and | log2FC| > 0.5 as a guideline for screening DEGs and DEMs, mRNAs (DEGs) and miRNAs (DEMs) were found to be differentially expressed. The volcano plot and heat map showed the expression patterns between the cancer and normal samples. The study followed the TCGA guidelines.

### GEO Database Validation and Survival Analysis

Two distinct data sets were acquired from the directory of the GEO (GSE17536, GSE29623)^2^ for validating the prognostic potential of genetic risk score. Three microarray expression profile datasets, GSE32323, GSE39582, and GSE74602, were also downloaded from the GEO database to validate the differentially expressed TFs.

The GSE17536 was submitted by Smith et al. (2010) and included 177 CRC patients. The GSE29623 was submitted by Chen et al. (2012) and included 65 CRC patients. The GSE32323, including 17 combos of carcinogenic and non-carcinogenic tissues from CRC subjects, was submitted by Khamas et al. (2012). The GSE39582 was submitted by Marisa et al. (2013) and included 566 CRC patients and 19 non-tumoral colorectal mucosa tissues. The GSE74602 was submitted by Pek et al. (2017) and included 30 CRC patients and 30 normal tissues.

### Cataloging of Transcriptional Factors

The Animal Transcription Factor DataBase (AnimalTFDB, ^3^) is a resource directed to come up with the most far-reaching and precise knowledge of transcription factors (Hu et al., 2019). A total of 1,665 transcription factors were downloaded from AnimalTFDB.

### Functional Enrichment Analysis

To investigated the prognostic function of the differentially expressed TFs, the Gene Ontology (GO) and Kyoto Encyclopedia of Genes and Genomes (KEGG) pathway enrichment analyses were carried out. Enrichment analyses for the DEGs were studied in the “clusterProfiler” package of R software (Green et al., 1988). Certain processes like biological process, cellular component, and molecular function have been wrapped by the results of the GO analysis. The standards used in the analysis were Gene Ontology terms and Kyoto Encyclopedia of Genes and Genomes pathways with a *P*-value < 0.05.

### Genetic Risk Score Model Construction

For more research and development of a genetic risk score, we chose the differentially regulated TFs first. To assess the connection between the expression of the differentially regulated TFs and their comprehensive durability, a univariate Cox proportional hazards regression analysis was carried out. The top ten important prognosis-related genes from the univariate Cox regression analysis were then considered candidate variables, and they were further investigated using a stepwise multivariate Cox proportional hazards regression analysis to predict each individual’s risk score (Lee et al., 2019).

### Copy Number Variation

The percentage of samples in which a particular RBP was amplified or deleted was calculated using the data from cBioPortal^4^.

### Methylation Data Analysis

Analysis of the level of methylation derived from the TCG database (Illumina Human Methylation 450 platform) was carried out *via* the linkedomics website^5^. The prediction of the CpG island of the ZNF26 promoter was carried out with MethPrimer^6^.

### Prediction of Gene Targets of Deregulated microRNAs in CRC

StarBase^7^ was used for prognosticating the gene targets of miRNAs.

### miRNA-miRNA, Protein-Protein Interactions Network Prediction

The miRNA−mRNA relationship, protein-protein interaction network was predicted using the CytoscapeVersion3.5.3 software.

### Patients and Samples

The CRC tissues and adjacent normal tissues were obtained from Xiangya Hospital of Central South University. This study was approved by the ethics committee of Xiangya Hospital. The patients were informed, and signed an informed consent form.

### Cell Lines and Cell Culture

The normal human colon mucosal epithelial cell line NCM460 and human colorectal cell lines HCT116, SW480, and SW620 used in this study were purchased from the American Type Culture Collection (ATCC; ^8^). The colorectal cell lines HCT8, Caco2, and RKO were kindly provided by Professor Wancai Yang (Key Laboratory of Precision Oncology of Shandong Higher Education, Institute of Precision Medicine, Jining Medical University). The NCM460, SW480, Caco2, HCT116, HCT8, SW620, and RKO cells were maintained in RPMI-1640 (Biological industries) with 10% FBS. All cells were cultured at 37°C with 5% CO_2_.

### Cell Transfection

Small interfering RNA (siRNA) targeting *ZNF26* was purchased from RiboBio (Guangzhou, China). The sequence of *ZNF26* siRNA is: *ZNF26* si#1: GAAGTCTCCATTCGTTGTA, *ZNF26* si#2: CTAGGAAGGCATCACTTCA. siRNA was transfected with jetPRIME DNA and siRNA transfection reagent (PolyPlus-transfection). Cells were harvested 48 h after transfection, and the knockdown efficiency was tested by quantitative reverse transcription polymerase chain reaction (qRT-PCR).

### RNA Extraction and Quantitative Reverse Transcription Polymerase Chain Reaction

Trizol reagent (Vazyme) was used for extracting the total RNA. Go Script reverse transcription system (Promega) was used for performing reverse transcription PCR. On the ABI Prism 700 thermal cycler, real-time qPCR was performed using GoTaq qPCR Master Mix (Promega). The following is the primer sequence: *ZNF26* (forward primer: TCAGGTCACAGCTCATTGTCCATC; reserved pr imer: CCACATTCGCTGCATTCATACGG); *CDK2* (forward pri mer: TGCCCTTTCACTGCCTATGG; reserved primer: GAGG AAAGCCAAGACCCACA); *CCNE2* (forward primer: GCCGA GCGGTAGCTGGTC; reserved primer: GGGCTGCTG CTTAGCTTGTAAA); *CDK6* (forward primer: ACGTGGT CAGGTTGTTT; reserved primer: TTTATGGTTTCAGTG GG); *CHEK1* (forward primer: TCATCCATTTCTAACA AATTCACTT; reserved primer: TGGGCTATCAATGGAA GAAAA); *GAPDH* (forward primer: CTGGGCTAC ACTGAGCACC; reserved primer: AAGTGGTCGTT GAGGGCAATG). *GAPDH* was used as an internal control.

### Cell Proliferation Assay

The cells (3,000/well) were cultured in a 96-well plate for 24 h, and the cell proliferation ability was investigated using the Cell Counting Kit 8 (CCK-8). For the colony formation assay, a 12-well plate containing 200–300 infected RKO cells was used. After 10–14 days of incubation, the cells were fixed with 4% paraformaldehyde, stained with crystal violet, photographed, and then counted. The EdU (RiboBio, Guangzhou, China) assay was performed according to the standard protocol. All experiments were repeated at least three times.

### Cell Cycle Analysis

After 48 h of specific interference with *ZNF26* in RKO cells with two siRNAs, the cells were collected and resuspended in 1 × PBS. The cells were then fixed in 70% ethanol overnight. The next day, after washing with PBS solution and centrifugation, the cells were stained with propidium iodide (PI) and analyzed by the FACSCalibur system (BD Biosciences, San Jose, California, United States).

### Statistical Analysis

We used R Studio (R version 3.5.2) for the statistical analysis. For univariate and multivariate analysis, SPSS version 20.0 was used. Graph Pad Prism 8.0 version for Windows was used to carry out the Kaplan Meier survival analysis. *P* < 0.05 was considered statistically significant.

## Results

### Integrated Genomic Analysis Reveals Genetic and Epigenetic Changes in TFs in Colorectal Cancer

**FIGURE 1:**
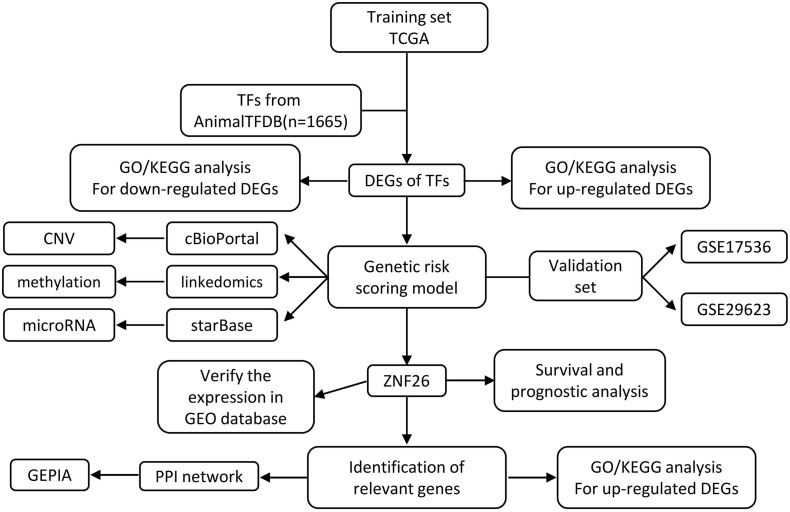
Schematic illustration of gene selection, data processing, and analysis of our study.

We obtained 1,665 transcription factors from Animal TFDB to elucidate different aspects of TF biology in CRC. To recognize the transcription factors that are modified in CRC due to the genetic and epigenetic effects, we performed an integrated bioinformatics analysis (Figure 1). Using these methods, we identified that TFs are differentially expressed in CRC. COX regression analysis was also performed to further investigate the relationship between the TFs and prognosis, namely, TFs were associated with CRC transformation and progression.

### Identification of the Differentially Regulated TFs and Functional Annotation

**FIGURE 2:**
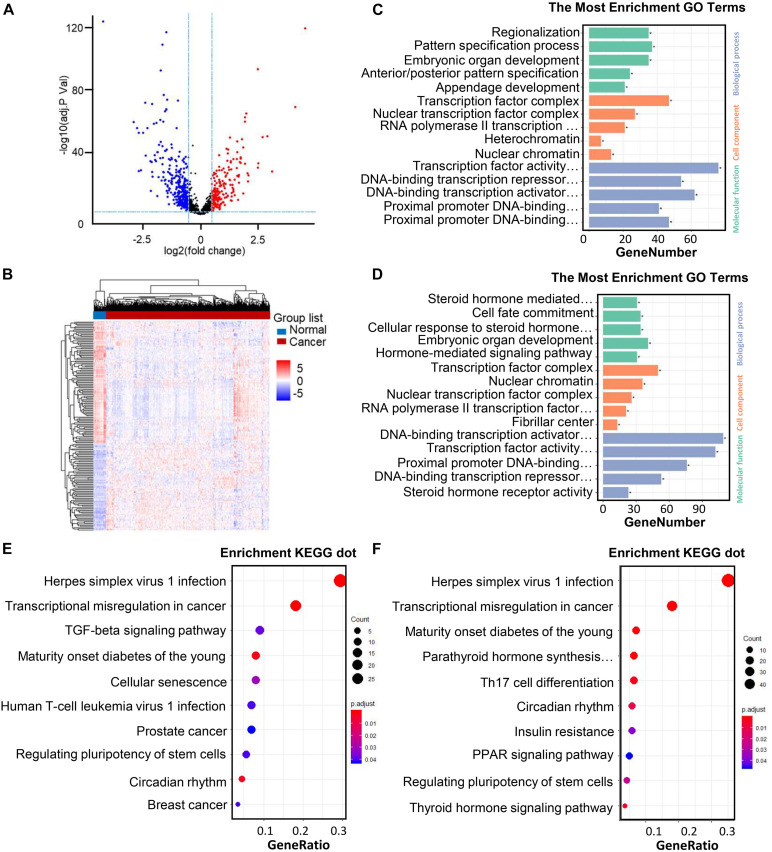
Differentially regulated TFs identified through TCGA datasets in CRC patients and the practical explanation of DEGs. **(A)** Volcano plot of the differentially regulated TFs between CRC and normal samples. The colored data thread shows the condition of TFs (red points: | log2FC| > 0.05 with FDR < 0.05; blue points: | log2FC| < 2 having FDR < 0.05). X-axis: | log2FC| (fold change); Y-axis: -log10 (adj. P Val) for each gene. **(B)** Heatmap of DEGs (210 upregulated and 316 downregulated genes). The development from low to high interpretation is shown by color in the heat maps moving from blue to red. FDR < 0.05, log2CPM > 1, and | log2FC| > 2 were set as the cut-off example. DEGs: differentially expressed genes; | log2FC| : log fold change; FC, fold change; CPM, numbering per million; FDR, the false discovery rate. **(C–D)** GO analysis and remarkably enhanced GO terms of upregulated genes **(A)** and downregulated genes **(B)**. **(E–F)** KEGG pathway carried out DEGs functional and signaling pathway enrichment. The improved items were analyzed by using gene counts, gene ratio, and adjusted p values to assess.

The Animal Transcription Factor DataBase (AnimalTFDB, see text footnote 3) is a resource aimed to have the most far-reaching and authentic knowledge for TFs (Hu et al., 2019). For further analysis, a total of 1,665 human TFs were identified from the AnimalTFDB. The “limma” package of the R software was used to evaluate the gene expression profiling of the TCGA samples, which included 568 CRC samples and 44 normal samples (Ritchie et al., 2015). A total of 525 TFs were differentially regulated between the normal tissues and CRC tissues. The volcano plot of each gene expression profile data has been shown in Figure 2A. A total of 210 progressive and 316 retrogressive DEGs were recognized in the CRC samples in contrast with the normal samples (Figure 2B).

We carried out the GO function and KEGG pathway to further investigate these differentially expressed TFs. The “cluster Profiler” package of the R software was used to examine the enrichment analyses for the upregulated and downregulated DEGs (Green et al., 1988).

In the biological process group, the progressive genes are principally intensified in regionalization, pattern specification procedure, embryonic organ development, anterior/posterior pattern specification as well as appendage development. As for the enrichment of retrogressive genes, steroid hormone intervenes with the signaling pathway, cell fate commitment, cellular feedback to steroid hormone stimulus, embryonic organ development, as well as embryonic organ development have been exhibited.

In another sort of analysis focusing on the GO cell components, upregulated transcription factors were mainly enriched in the transcription factors, nuclear transcription factor network, RNA polymerase II transcription factor network, heterochromatin, and nuclear chromatin, while the downregulated TFs are enriched in the transcription factor complex, nuclear chromatin, nuclear transcription factor network, RNA polymerase II transcription factor complex, and fibrillar center.

Besides, in the GO cell function analysis, upregulated TFs are significantly enriched in the transcription factor activity, DNA-binding transcriptional repressor activity, DNA-binding transcriptional activator activity, proximal promoter DNA-binding transcription repressor activity, and proximal promoter DNA-binding transcription activator activity. Meanwhile, downregulated TFs are enriched in the DNA-binding transcriptional activator activity, transcription factor activity, proximal promoter DNA-binding transcription, DNA-binding transcription repressor activity, and DNA-binding transcription repressor activity (Figures 2C,D). These significantly enriched GO terms can aid in having a better understanding on the roles of these differentially regulated TFs in the initiation and progression of CRC.

At the same time, the KEGG pathway analysis was carried out on the up- and downregulated TFs. Upregulated TFs have enriched in herpes simplex virus type 1 infection, transcriptional misregulation in cancer, TGF-β signaling pathway, maturity onset diabetes of the young, and Cellular senescence, while downregulated TFs are enriched in herpes simplex virus type 1 infection, transcriptional misregulation in cancer, maturity onset diabetes of the young, parathyroid hormone synthesis, secretion, and action, Th17 cell differentiation (Figures 2E,F).

### Genetic Risk Score Model Construction

**FIGURE 3:**
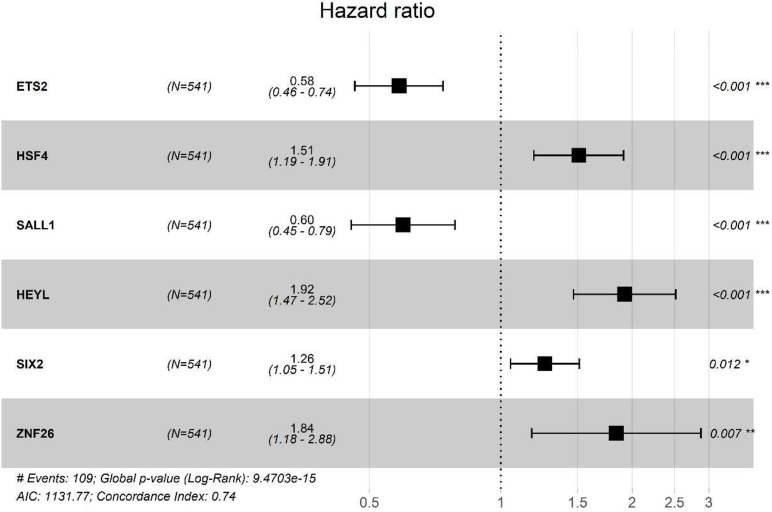
Multivariate Cox proportional hazards retrogression investigation of the significant prognostic TFs genes. CI, confidence interval; *p*, *p*-value; No. at risk, number of patients already exposed. Prognostic value of genetic risk scores and prediction of cancer survival rate in combination with clinical factors.

From the prognostic perspective, we further studied the differentially regulated TFs in CRC in the TCGA-CRC training dataset. The top 10 significant prognostic genes, *ETS2*, *LBX2*, *HSF4*, *SALL1*, *SIX4*, *HEYL*, *POU6F1*, *SIX2*, *ZNF26*, *and TLX2*, were sought out by a univariate Cox regression (*p* < 0.001) (Table 1). To further select the TFs with the highest prognostic value, a multivariate Cox regression analysis was performed for investigating its impact, and six hub TFs were selected for establishing a risk model for CRC patients, including *EST2*, *HSF4*, *SALL1*, *HEYL*, *SIX2*, and *ZNF26*. The hazard ratios of the four genes, *HSF4*, *HEYL*, *SIX2*, and *ZNF26*, were positive, which show their negative match with the prognosis. For *ETS2* and *SALL1*, however, the ratios were negative, indicating that they might be positively related to the prognosis (Figure 3). The following genetic risk score model was used to calculate the risk score of each patient:

$$\begin{array}{l} Genetic\;Risk\;score\;=\;(-0.53718)\;\times \;ETS2\;+\;0.411836\;\\ \times \;HSF4\;+\;(-0.51552)\;\times \;SALL1\;+\;0.653934\;\times \;HEYL\;\\ +\;0.233067\;\times \;SIX2\;+\;0.610575\;\times \;ZNF26 \end{array}$$

**TABLE 1:** Top 10 notable prognostic genes disclosed by univariate Cox proportional hazards regression.

Gene	Hazard Ratio (HR)	95% CI of HR	P-value
*ETS2*	0.6084	(0.4785–0.7735)	0
*LBX2*	1.9164	(1.3474–2.7256)	0.0003
*HSF4*	1.4551	(1.1822–1.7910)	0.0004
*SALL1*	0.6147	(0.4689–0.8057)	0.0004
*SIX4*	1.4924	(1.1824–1.8838)	0.0008
*HEYL*	1.5239	(1.1825–1.9638)	0.0011
*POU6F1*	1.8114	(1.2650–2.594)	0.0012
*SIX2*	1.3201	(1.1159–1.5618)	0.0012
*ZNF26*	1.8475	(1.2652–2.698)	0.0015
*TLX2*	1.9828	(1.2931–3.0403)	0.0017

**ETS2*, ETS proto-oncogene 2; *LBX2*, adybird homeobox 2; *HSF4*, heat shock transcription factor 4; *SALL1*, spalt like transcription factor 1; *SIX4*, SIX homeobox 4; *HEYL*, hes related family bHLH transcription factor with YRPW motif like; *POU6F1*, POU class 6 homeobox 1; *SIX2*, SIX homeobox 2; *ZNF26*, zinc finger protein 26*; TLX2*, T cell leukemia homeobox 2.*

**FIGURE 4:**
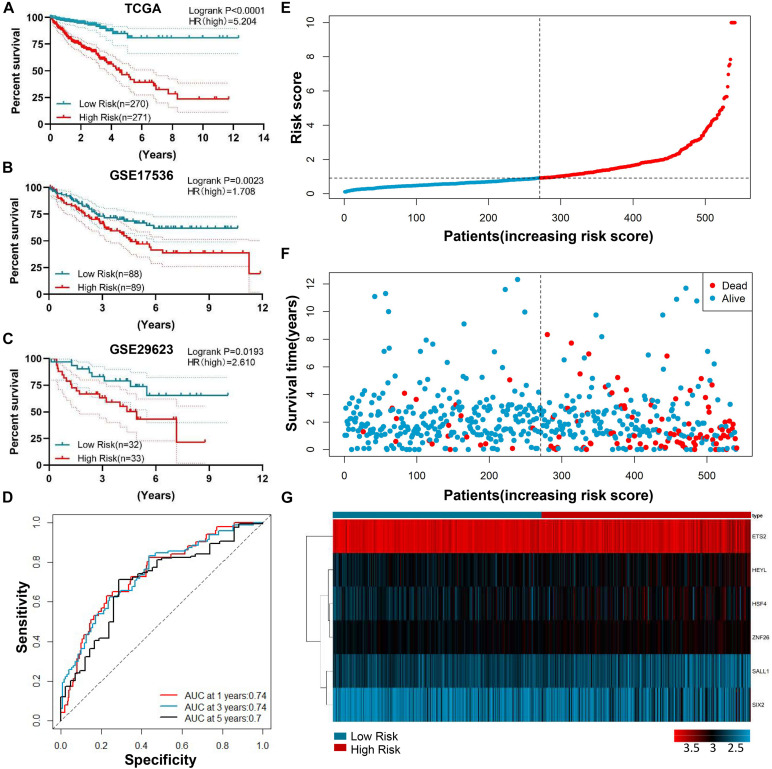
Prognostic value of genetic risk scores and an indication of the cancer survival rate in combination with clinical factors. **(A)** Prognostic significance of the median genetic risk score 2 group risk model (Low vs. High) with a significant difference in the TCGA-CRC training dataset. **(B–C)** The Kaplan-Meier curves concerning the overall survival of CRC in GSE17536 **(B)** and GSE29623 **(C)** validation cohort. **(D)** The survival time-dependent ROC curve. The AUC of the 1 year, 3 years, and 5 years ROC curve were 0.74, 0.74, and 0.7. AUC: area under the curve; ROC: Receiver operating characteristic. **(E–G)** The division of the six-gene signature, survival distinction, and interpretation portrait of the six genes of patients in the training data set.

Based on the median risk score, the CRC patients with risk scores higher than the median were defined as a high-risk group, and risk scores lower than the median were defined as a low-risk group in the TCGA-CRC training dataset. The Kaplan-Meier curve showed that the patients in the high-risk group had a worse prognosis compared to those in the low-risk group (*P* < 0.001) (Figure 4A). To verify whether the six TFs model exhibited a similar predictive value in the other CRC cohorts, a similar formula was used to generate the risk score of each patient in the two OS validation datasets (GSE17536 and GSE29623). The results showed significant prognostic values were *p* = 0.0023 (Figure 4B) and *p* = 0.0193 (Figure 4C), respectively. The AUC of the 1 year, 3 years, and 5 years ROC curves were 0.74, 0.74, and 0.7, respectively, which advocated that our risk model was accurate in the forecasting of survival in the testing data set (Figure 4D). The assigning of risk score, survival status, and the interpretation of six genes were also predicted for each patient (Figures 4E–G).

To further confirm the prognostic role of the genetic risk score mode in CRC patients, we performed the univariate and multivariate survival analysis. Univariate analysis identified eight prognostic factors: ages (≥60 vs. <60), T (T3–T4 vs. Tis–T2), N (N1–2 vs. N0), M (M1–2 vs. M0), Stage (III–IV vs. I–II), Kras mutation (mut vs. wt), CEA level (≥5 vs. <5 μg/ml), and Risk (high risk vs. low risk). Multivariate survival analysis further uncovered that age (*p* = 0.002), M stage (*p* = 0.044), TNM stage (0.017), CEA level (0.044), and the genetic risk score (<0.001) were separately prognostic of OS (Table 2).

**TABLE 2:** Univariate and multivariate investigation of clinic pathologic aspects for its comprehensive survival in Colorectal Cancer patients.

Risk factors	Univariate investigation		Multivariate investigation	
	Hazard Ratio (95% CI)	*P*-value	Hazard Ratio (95% CI)	*P*-value
Gender (male vs. female)	0.913 (0.625–1.333)	0.636		
Age (≥60 vs. <60)	1.681 (1.068–2.467)	0.025*	3.441 (1.576–7.515)	0.002**
T (T3–T4 vs. Tis–T2)	2.248 (1.170–4.319)	0.015*	1.378 (0.475–3.995)	0.555
N (N1–2 vs. N0)	2.811 (1.899–4.160)	<0.001***	0.306 (0.083–1.130)	0.076
M (M1–2 vs. M0)	4.529 (2.974–6.897)	<0.001***	2.224 (1.021–4.845)	0.044*
Stage (III–IV vs. I–II)	2.991 (1.981–4.514)	<0.001***	6.434 (1.398–29.616)	0.017*
Kras mutation (mut vs. wt)	1.951 (1.252–3.040)	0.003**	1.249 (0.521–2.993)	0.618
CEA level (≥5 vs. <5 μg/ml)	2.773 (1.646–4.672)	<0.001***	1.900 (1.018–3.545)	0.044*
Risk (high risk vs. low risk)	5.201 (3.170–8.533)	<0.001***	4.637 (2.222–9.680)	< 0.001***

CI, confidential interval, *p < 0.05; **p < 0.01; ***p < 0.00.

### Mechanism of Abnormal Expression of TFs in CRC

**FIGURE 5:**
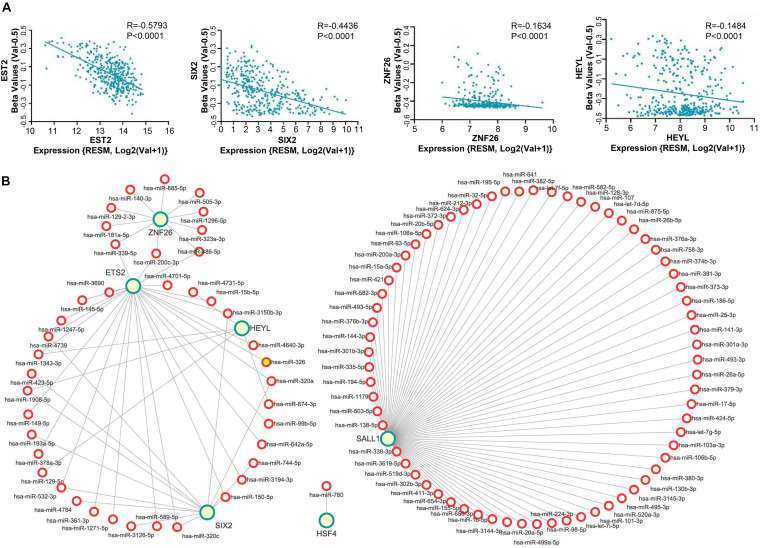
Mechanism of abnormal expressed TFs in CRC. **(A)** Expression boxplots of gene *ETS2*, *SIX2*, *ZNF26*, and *HEYL* were conflictingly associated with DNA methylation. **(B)** The targeting relationship between the differentially expressed miRNAs and six TFs were identified using the starBase and a miRNA-gene correlation network which was constructed using the Cytoscape software. Green circles indicate the six TFs, red circle indicates the predicted miRNA. The length of the lines indicates correlation strength.

To further investigate the reason why these six TFs are expressed differently in CRC, we analyzed three possibilities, for instance: copy number variation, methylation, and mircoRNA. First, we analyzed the copy number variation from the TCGA database through the cBioPortal^9^. As for these six genes, the variation of copy number among CRC patients was not remarkable (Supplementary Material 1A). Meanwhile, analysis of the TCGA database using the linkedomics website (see text footnote 5) revealed that the expression of *ETS2*, *SIX2*, *ZNF26*, and *HEYL* were negatively correlated with DNA methylation (Figure 5A), while expression of the *SALL1* and *HSF4* were not (Supplementary Material 1B).

Since miRNAs play a role in RNA silencing or degradation and gene expression regulation after transcription by base-pairing with complementary sequences within mRNA molecules and then regulating gene expression, we conducted the following research. First, we found that 321 microRNAs were upregulated and 312 were downregulated through the TCGA database for miRNA in CRC (Supplementary Material 1C). Then, the differentially expressed miRNAs contribute to predicting the targeting relationship with six TFs by using the starBase (see text footnote 7). As shown in Figure 5B, the network of miRNA and six TFs interactions were visualized in Cytoscape. The downregulated miRNAs in CRC are associated with the four upregulated transcription factors, namely, *HSF4*, *HEYL*, *SIX2*, and *ZNF26*. The upregulated miRNAs in CRC are associated with the downregulated transcription factors, namely, *SALL1* and *ETS2*.

### *ZNF26* Is Linked With the Poor Outcomes of CRC Patients

**FIGURE 6:**
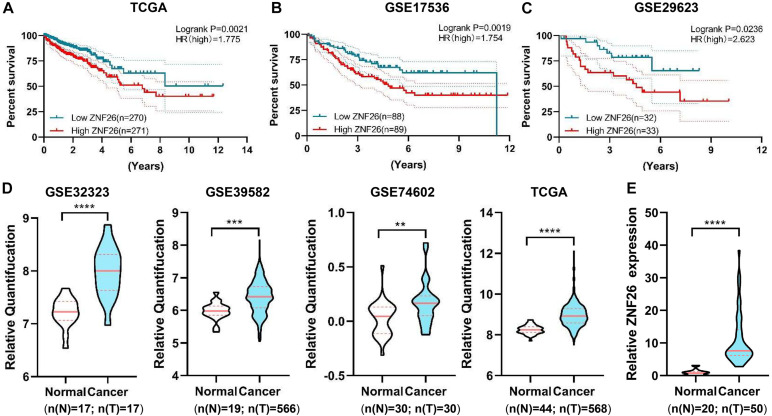
*ZNF26* is associated with poor outcomes in CRC patients. **(A–C)** High *ZNF26* interpretation is associated with low survival of CRC patients. The area indicates results of Kaplan-Meier analysis of patients’ life survival on a cohort of TCGA **(A)**, GSE17536 **(B)**, GSE29623 **(C)**. The patients with high vulnerability and low vulnerability are characterized by red and blue lines, respectively. Each group having the numbers of patients are shown under graphs. **(D)** Violin plot shows the normal and tumor expression ratios of *ZNF26* in GSE32323, GSE39582, GSE74602, and TCGA. The numbers of patients in each group are shown below the graphs. **(E)** qRT-PCR analysis of *ZNF26* in 20 normal colorectal and 50 CRC samples.

After a validation and survival analysis on these six genes in GSE29623 and GSE17536, only *ZNF26* with a high expression was found to be associated with the poor prognosis in patients (Figures 6A,B). The results showed that, in the TCGA database, *ZNF26* was linked with the poor prognosis of patients (Figure 6C). To study the expression of *ZNF26* in CRC tissues in detail, *ZNF26* was found to be progressive in CRC through the TCGA database and GEO database analysis (GSE32323, GSE39582, and GSE74602) (Figure 6D). Next, we further verified the expression level of *ZNF26* in CRC tissue samples by qRT-PCR. The results showed that, compared with adjacent normal intestinal mucosa tissues, the mRNA level of *ZNF26* was significantly upregulated in CRC tissue (Figure 6E).

### *ZNF26* Is Positively Correlated With Cell Cycle-Related Proteins (CDK2, CCNE2, CDK6, and CHEK1)

To figure out the mechanism of *ZNF26* function in CRC, GO analysis and KEGG analysis were performed on *ZNF26* positively/negatively correlated (| R| > 0.3) genes.

Gene Ontology analysis revealed that the genes that are positively associated with *ZNF26* were mainly enriched in the detection of the chemical stimulus involved in sensory perception, DNA conformation change, and chromosomal region, while the genes that are negatively associated with *ZNF26* were mainly enriched in neutrophil-mediated immunity, neutrophil activation, and cell adhesion molecule binding (Supplementary Materials 2A,B).

Besides, through the KEGG pathway analysis, the genes that are positively associated with *ZNF26* are enriched in Herpes simplex virus 1 infection, Olfactory transduction, and Ubiquitin mediated proteolysis, while the negatively related genes are enriched in Thermogenesis, Huntington’s disease, and Alzheimer’s disease (Supplementary Materials 2C,D).

**FIGURE 7:**
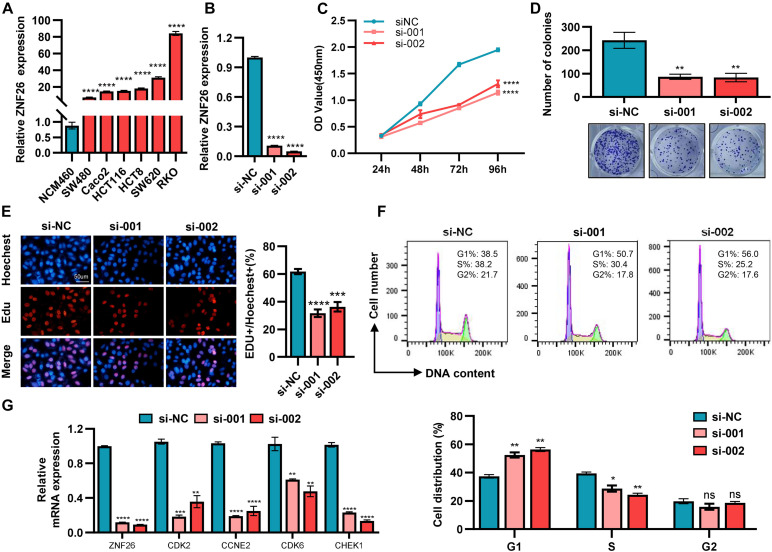
*ZNF26* promotes cell proliferation and is positively correlated with cell cycle-related proteins. **(A)** The expression of *ZNF26* was analyzed by qRT-PCR in NCM460 and CRC cell lines. **(B)** The knockdown efficiency of *ZNF26* in RKO cells was detected by qRT-PCR. **(C–E)** The capability of cell proliferation was determined by CCK8 assay **(C)**, colony formation assay **(D)**, and EdU assay **(E)** with *ZNF26* knockdown. Scale bar: 50 μm. **(F)** Distribution of cells in three cell cycle phases of RKO was examined by flow cytometry assay, and the graph shows the quantification for each phase. **(G)** qRT-PCR analyzed the mRNA levels of CDK2, CCNE2, CDK6, and CHEK1 after knocking down *ZNF26* in RKO.

The abnormality of the cell cycle played a major role in the pathogenesis of CRC (Gan et al., 2018). The mRNA expression of *ZNF26* in colorectal cancer cell lines was detected by qRT-PCR (Figure 7A). We found that *ZNF26* was highly expressed in the CRC cell lines, as compared with normal intestinal epithelial cells NCM460. Then, we used *ZNF26* siRNA for specific silencing, and qRT-PCR to detect the transfection efficiency (Figure 7B). We explored the cell proliferation ability through CCK8 (Figure 7C) and cell plate cloning (Figure 7D). The analysis showed that knocking down *ZNF26* significantly inhibited the proliferation and colony formation of RKO cells. The EdU experiment also confirmed the proliferation-promoting effect of *ZNF26* in the CRC cell lines (Figure 7E). Overall, these findings provide evidence that knocking down *ZNF26* can inhibit the proliferation of CRC cells. To evaluate the possible role of *ZNF26* in the regulation of the cell cycle (Figure 7F), we analyzed the phase distribution of the cell cycle by a flow cytometric analysis. It was observed that the knockdown of *ZNF26* efficiently brought about an increase in cell percentage in the G1 phase, and a decrease in cell percentage in the S phase.

Moreover, the TCGA database showed that *ZNF26* was positively correlated with the cell cycle-related genes (Supplementary Material 2E). We also evaluated the cell cycle-related genes by the STRING online database and Cytoscape software for selecting the most effective ones (Supplementary Material 2F). In addition, the correlation between the Top4 gene and *ZNF26* analyzed by the GEPIA database indicated that *ZNF26* was positively correlated with CDK2, CCNE2, CDK6, and CHEK1 (Supplementary Material 2G). At the same time, after knocking down *ZNF26* in the RKO cell line, the expression of these four genes was detected by qRT-PCR. The results showed that the knockdown of *ZNF26* can significantly downregulate the expression of CDK2, CCNE2, CDK6, and CHEK1 (Figure 7G).

## Discussion

Colorectal cancer is the third most common cancer in the world and the fourth highest cancer mortality (Torre et al., 2015; Connell et al., 2017). Despite the advances in CRC therapy through medical technology, surgical techniques, and chemotherapy, there has been no effective diagnostic method to monitor the tumor progression or recurrence (Issa and Noureddine, 2017; Yang et al., 2019). Recent studies demonstrated that TFs play a vital role in the spread of cancer, and transcription factor dysregulation can lead to malignant growth and the development of cancer (Daly, 2017; Songdej and Rao, 2017; Cao et al., 2018). However, few studies have focused on the characteristics of TFs in CRC. In this study, the expression of TFs and their significant role in the growth and progression of CRC were described, and a genetic risk scoring model was established.

In our analysis, we found 526 differentially regulated TFs in the CRC samples; of which, 40% were upregulated. Both the progressive and retrogressive TFs were explored in the Gene Ontology term enrichment analysis and Koyoto Encyclopedia of Genes and Genomes pathway analysis to provide a practical explanation. GO and KEGG analyses also showed that 526 differentially regulated TFs were significantly enriched in various cancer-related functions and pathways. These findings can aid our understanding of how transcription factors promote/suppress CRC.

We identified six prognostic-related transcription factors using the 526 differentially expressed TFs described above for the COX regression analysis. The genetic risk scoring model’s predictive ability was confirmed by the significant differences between the survival curves of the high-risk and low-risk groups shown in the genetic risk scoring model. Building on the GSE29623 and GSE17536 data sets, our genetic risk scoring model was validated to be versatile. ROC curve analysis also revealed that the differentially expressed genes could be an excellent early prediction on survival prognosis.

The combination of the mRNA expressions of *HSF4*, *HEYL*, *SIX2*, *ZNF26*, *ETS2*, and *SALL* was the basis of the genetic risk scoring model. *SALL1*, *HSF4*, *SIX2*, *ETS2*, and *HEYL* have been reported in tumors. For instance, *SALL1* acted as a novel biomarker for the prognosis of early-stage head and neck cancer (Misawa et al., 2018). *HSF4* could be used as a prognostic marker in CRC (Yang et al., 2017), while *SIX2* might be a diagnostic and prognostic biomarker in Wilms’ tumor (Song et al., 2015). Also, the high expression of *ETS2* predicted a poor prognosis in severe myeloid leukemia (Fu et al., 2017). Among them, *ZNF26*, *SALL1*, *HEYL*, and *SIX2* have not yet been reported in CRC. Therefore, due to the identification and verification of the prognostic factors that can improve the treatment (Zhang et al., 2018b), we wonder to find out the underlying mechanisms of abnormally expressed TFs in CRC. Out of this, we have explored multiple reasons for the abnormal expression of TFs in CRC, including gene-level (Daly, 2017), methylation level (Bonder et al., 2017), and miRNA-mediated regulation (Zhang et al., 2018a). In cases where changes into TFs (including mutations) are known to cause cancer and other diseases, we analyzed the possible mutation sites of these TFs in CRC and found that the six TFs mutations (including deletions and amplifications) are not obvious. Changes in gene methylation may have a critical impact on gene expression (Bhargava et al., 2017). In our study, we found that *ETS2*, *SIX2*, *ZNF26*, *HEYL* were negatively correlated with DNA methylation. We used the six prognostic-related TFs to establish a common regulatory network with miRNAs in CRC where the expression of miRNAs was analyzed.

Based on GSE29623, GSE17536, GSE32323, GSE39582, and GSE74602, validating the expression and survival analysis of these six TFs proceeded. It was shown that *ZNF26* was progressive in CRC, and its excessive interpretation foreboded the deficient prediction. The huge transcription factor family in the human genome is the zinc finger protein family; of which, *ZNF26* is a prominent member (Jen and Wang, 2016). New studies show that the abnormal expression of ZNF proteins come up with tumorigenesis in different tumors, such as CRC (Yang et al., 2008), lung cancer (Lo et al., 2012), and ovarian cancer (Aslan et al., 2015). Currently, there are few studies on *ZNF26*, especially in tumors, which have not been reported yet. In this study, we confirmed that the expression level of ZNF26 in CRC tissues and cells is significantly upregulated, and knocking down ZNF26 can effectively inhibit cell proliferation. With biological prediction, it was shown that *ZNF26* is positively correlated with cyclins (CDK2, CCNE2, CDK6, and CHEK1). More studies are needed to investigate the potential of the CRC-related TFs, especially *ZNF26*, for diagnosis, prognosis, and targeted therapy.

In conclusion, our research developed a TFs signature through a series of bioinformatics analyses, which proved to be an accurate independent prognosticator in CRC. Concurrently, we comprehensively view the six abnormally regulated TFs in CRC and mechanisms which may result in this abnormal regulation. Moreover, the elevated expression of *ZNF26* has been demonstrated in CRC indicating a poor prognosis of patients. Hence, we tested the hypothesis that, as a novel biomarker, *ZNF26* might be potentially valuable in the diagnosis and prognostic evaluation for colorectal cancer.

## Data Availability Statement

The datasets presented in this study can be found in online repositories. The names of the repository/repositories and accession number(s) can be found in the article/Supplementary Material.

## Author Contributions

JL and YL designed the research studies and analyzed the data. JL wrote the manuscript. YG, QX, RT, and GS contributed to check the figures and manuscript. GY supervised the project. All authors read and approved the final manuscript.

## Conflict of Interest

The authors declare that the research was conducted in the absence of any commercial or financial relationships that could be construed as a potential conflict of interest.

## Footnotes

1 https://portal.gdc.cancer.gov/

2 http://www.ncbi.nlm.nih.gov/geo/

3 http://bioinfo.life.hust.edu.cn/AnimalTFDB

4 http://www.cbioportal.org/

5 http://www.linkedomics.org/login.php

6 http://www.urogene.org/methprimer/

7 http://starbase.sysu.edu.cn/index.php

8 http://www.atcc.org/

9 https://www.cbioportal.org/
