# Supplementary material for: Identification of ZNF26 as a Prognostic Biomarker in Colorectal Cancer by an Integrated Bioinformatic Analysis
Source: Front Cell Dev Biol. 2021 Jun 11;9:671211. doi: 10.3389/fcell.2021.671211 (PMC8226143; doi:10.3389/fcell.2021.671211)
Supplement: Supplementary file 1 [file Table_1.DOCX]

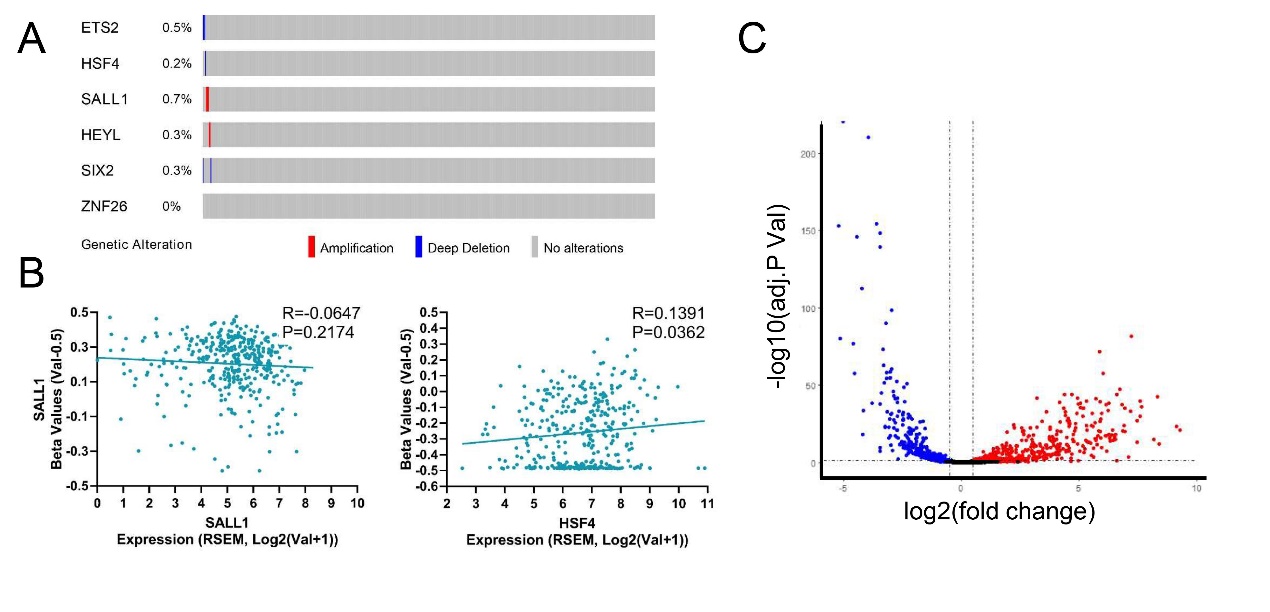


**Supplemental 1 Mechanism of abnormal expressed TFs in CRC.**

A. The copy number variation of gene *ETS2*, *HSF4*, *SALL1*, *HEYL*, *SIX2*, *ZNF26* from TCGA database were not remarkable.

B. Expression boxplots of gene *SALL1* and *HSF4* were not correlated with DNA methylation.

C. Volcano plot of the differentially regulated microRNAs between CRC samples and normal samples. The color of the data plots represents the status of TFs (red points: |log2FC|>0.05 with FDR <0.05; blue points: |log2FC|<2 with FDR <0.05). X-axis: |log2FC| (log fold change); Y-axis: -log10 (adj. P Val) for each gene.


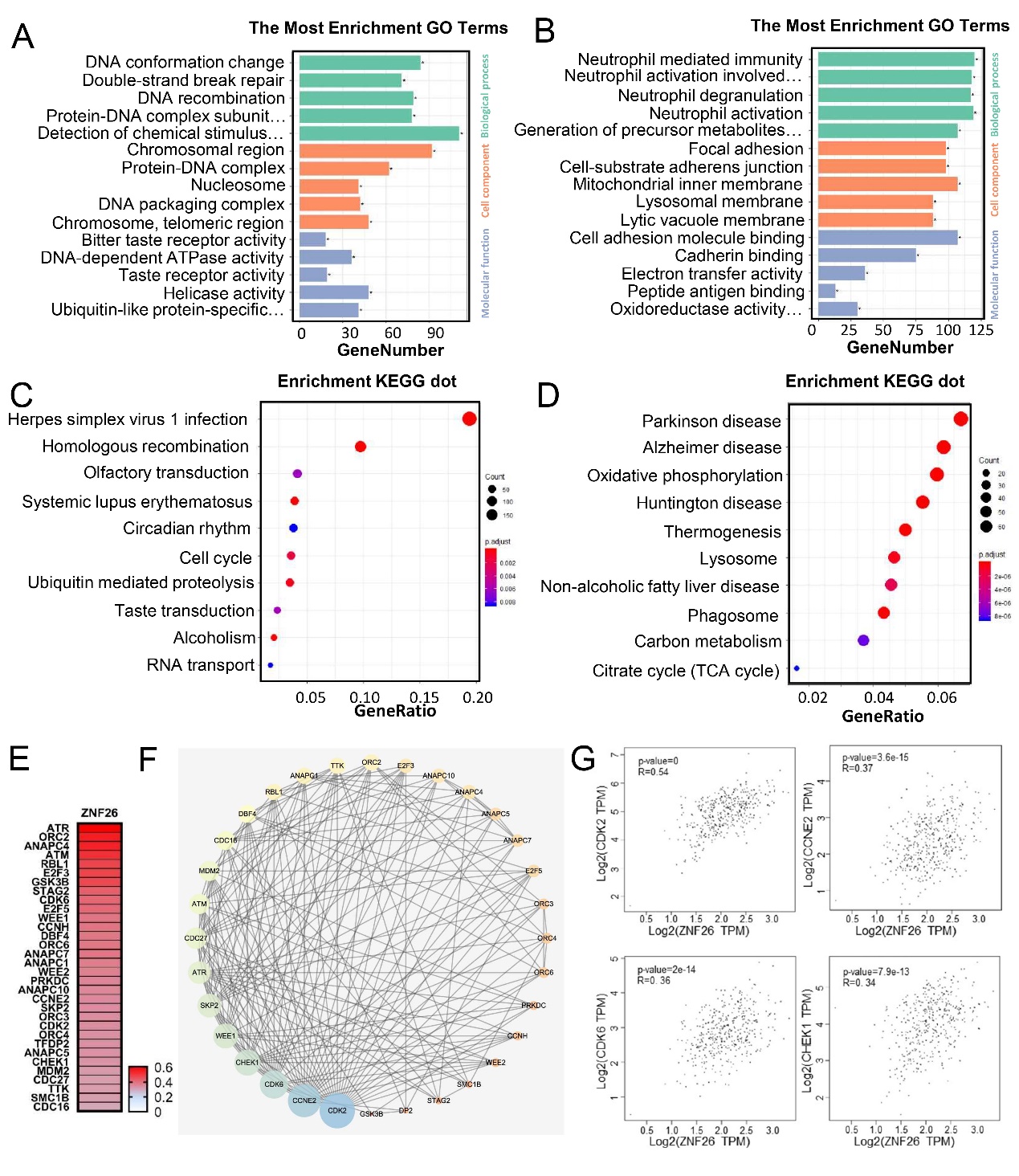


**Supplemental 2: Mechanism of *ZNF26* function in CRC.**

A-B. GO analysis and significant enriched GO terms of the genes positively associated with *ZNF26* genes (A) and the genes negatively associated with *ZNF26* (B).

C-D. KEGG pathway analysis of genes positively associated with *ZNF26* (C) and the negatively related genes (D). Three parameters including gene ratio, gene counts, and adjusted p value were used to evaluate the enriched items.

(E) Hot map show *ZNF26* was positively correlated with cell cycle-related genes in TCGA.

(F) By practicing STRING online database and Cytoscape software cell cycle-associated genes were clarified into PPI network complex.

(G) Correlation expression of *ZNF26* with the cell cycle-related genes using GEPIA.
